# Supplementary material for: Robust learning from noisy, incomplete, high-dimensional experimental data via physically constrained symbolic regression
Source: Nat Commun. 2021 May 28;12:3219. doi: 10.1038/s41467-021-23479-0 (PMC8163752; doi:10.1038/s41467-021-23479-0)
Supplement: Supplementary file 2 — Description of Additional Supplementary Files [file 41467_2021_23479_MOESM2_ESM.docx]

Description of Additional Supplementary Files

Title: Supplementary Movie 1

Description: A video which shows a qualitative comparison between the Helmholtz-reconstructed pressure field and the vorticity field time evolution.
